# FeMOFs/CO loading reduces NETosis and macrophage inflammatory response in PLA based cardiovascular stent materials

**DOI:** 10.1093/rb/rbae140

**Published:** 2024-12-03

**Authors:** Yinhong Xie, Mengchen Chi, Xinlei Yang, Ruichen Dong, Ao Yang, Antao Yin, Yajun Weng

**Affiliations:** Key Laboratory of Advanced Technologies of Materials, Ministry of Education, Southwest Jiaotong University, Chengdu 610031, China; School of Materials Science and Engineering, Southwest Jiaotong University, Chengdu 610031, China; Key Laboratory of Advanced Technologies of Materials, Ministry of Education, Southwest Jiaotong University, Chengdu 610031, China; School of Materials Science and Engineering, Southwest Jiaotong University, Chengdu 610031, China; Key Laboratory of Advanced Technologies of Materials, Ministry of Education, Southwest Jiaotong University, Chengdu 610031, China; School of Materials Science and Engineering, Southwest Jiaotong University, Chengdu 610031, China; Institute of Biomedical Engineering, College of Medicine, Southwest Jiaotong University, Chengdu, Sichuan 610031, China; Key Laboratory of Advanced Technologies of Materials, Ministry of Education, Southwest Jiaotong University, Chengdu 610031, China; Institute of Biomedical Engineering, College of Medicine, Southwest Jiaotong University, Chengdu, Sichuan 610031, China; Key Laboratory of Advanced Technologies of Materials, Ministry of Education, Southwest Jiaotong University, Chengdu 610031, China; Institute of Biomedical Engineering, College of Medicine, Southwest Jiaotong University, Chengdu, Sichuan 610031, China; Key Laboratory of Advanced Technologies of Materials, Ministry of Education, Southwest Jiaotong University, Chengdu 610031, China; Institute of Biomedical Engineering, College of Medicine, Southwest Jiaotong University, Chengdu, Sichuan 610031, China; Key Laboratory of Advanced Technologies of Materials, Ministry of Education, Southwest Jiaotong University, Chengdu 610031, China

**Keywords:** FeMOFs, carbon monoxide, NETosis, macrophage, inflammation

## Abstract

Modification of polylactic acid (PLA) is a promising strategy for the next generation of bioresorbable vascular stent biomaterials. With this focus, FeMOFs nanoparticles was incorporated in PLA, and then post loading of carbon monoxide (CO) was performed by pressurization. It showed FeMOFs incorporation increased hydrophilicity of the surface and CO loading, and CO release was sustained at least for 3 days. It is well acknowledged NETosis and macrophage mediated inflammation are the principal effectors of atherosclerosis and cardiovascular disease, and it further increases the risk of late stent thrombosis and restenosis. In this study, the effects of CO release of PLA/FeMOFs/CO on NETosis and macrophage behavior were thoroughly explored. *In vitro* evaluation results showed that PLA/FeMOFs/CO significantly inhibited neutrophil extracellular traps (NETs) release and neutrophil elastase expression by reducing intracellular reactive oxygen species in a simulated inflammatory environment. It reduced Lipopolysaccharide-induced macrophage inflammation with decreased tumor necrosis factor-α expression and increased IL-10 expression. Meanwhile it enhanced endothelial cell activity and growth in inflammatory environment, and inhibited platelet adhesion and activation. *In vivo* implantation results confirmed that PLA/FeMOFs/CO reduced the macrophages and neutrophils mediated inflammatory response, thus reduced the neointimal hyperplasia. Overall, PLA/FeMOFs/CO effectively prevented the inflammation and restenosis associated with PLA implantation. Our study provides a new strategy to improve the immunocompatibility of PLA implant materials.

## Graphical abstract

**Figure:**
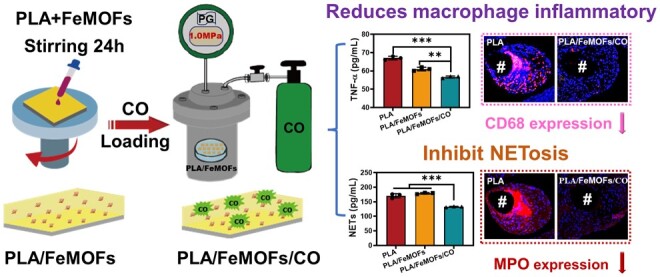

## Introduction

Cardiovascular diseases, known as atherosclerosis, is the leading cause of mortality and morbidity throughout the world [1]. Stent intervention, which can rapidly restore the blood flow, is currently the main method for the treatment of atherosclerosis [2]. There are long-term risks of non-degradable vascular stent which permanently retains *in vivo* as foreign body [3, 4]. Recently, the development of bioresorbable stent is expected to resolve the problem of permanent retention and late adverse events. Polylactic acid (PLA) is a biodegradable polymer widely used in biomedical applications due to its hydrolysis under physiological conditions and relatively good biocompatibility [5]. As the first bioresorbable stent to be used in clinic, several studies have confirmed the safety and efficacy of PLA stent in cardiovascular patients [6]. But its hydrophobicity and inherent inflammatory response also lead to complications such as thrombosis, restenosis and fibrosis [7, 8]. Titanium ions, Tantalum dioxide nanoparticles and composite dopamine coatings were used for increasing the hydrophilicity of PLA stents, and it seemed that this modification somehow increased biocompatibility [9–11]. However, there is still a lack of material related intervention of the atherosclerotic inflammatory microenvironment and thus regulation of inflammatory cell behavior.

Neutrophils are the first responders of inflammatory response and NETosis, a form of neutrophil-related cell death, plays an essential role in the pathogenesis of various inflammatory diseases such as atherosclerosis. NETosis differs from necrosis and apoptosis, and the induction of NETosis by various stimuli depends on reactive oxygen species (ROS) produced by nicotinamide adenine dinucleotide phosphate (NADPH) oxidase [12]. The extrusion of neutrophil extracellular traps (NETs) is accompanied by NETosis. NETs are meshwork structures consisting primarily of a DNA skeleton and decorated with neutrophil elastase (NE), myeloperoxidase (MPO) and histones (H3) [13]. NETs can induce endothelial dysfunction, trigger coagulation, and have been detected in atherosclerotic and thrombotic lesions in both humans and mice [14–17]. Moreover, NETs can trigger proinflammatory immune responses [18] and increase the risk of in-stent restenosis and thrombosis. Notably, researches in the field of cardiovascular materials mainly focused on inhibiting platelet activation, inhibiting smooth muscle cell proliferation and promoting endothelialization [19, 20]. Only a few focused on the influence of macrophage behavior [21], while few attentions were paid to the regulation of neutrophil behavior especially for NETosis or NETs.

Carbon monoxide (CO) is attracting increasing attentions due to its role as a gasotransmitter with cytoprotective and inflammation regulating properties. Endogenous CO is produced primarily by heme-catalyzed heme oxygenase, which is involved in physiological and pathological processes [22]. It was reported that CO has a variety of functions, including vasodilator, antiplatelet aggregation and activation, antiapoptotic, anti-inflammatory and antioxidant [23–27]. Some studies showed the effectiveness of CO in the cardiovascular disease therapy [28, 29]. However, it is still a challenge of loading and sustained release of CO. Moreover, the effects of CO on NETosis remain to be explored.

**Figure 1.**
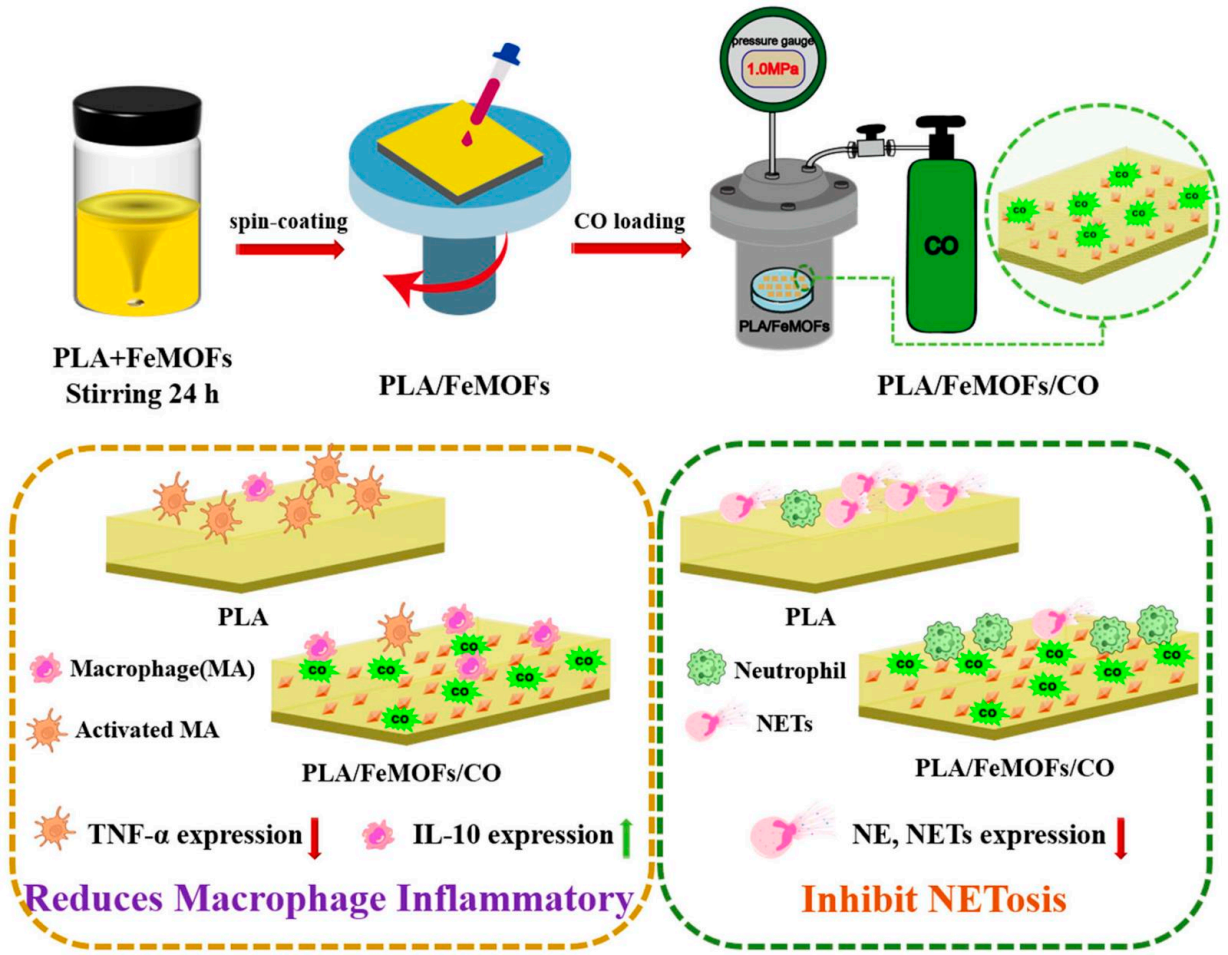
Schematic illustration of PLA/FeMOFs/CO for reducing NETosis and macrophage inflammatory.

In our previous study, FeMOFs nanoparticles were selected as the carrier of CO under normal pressure, which showed good affinity of CO and prolonged the CO release duration [30]. Based on the above considerations, in this study FeMOFs was incorporated in PLA and CO was loaded by a post-loading strategy (Figure 1). The role of CO release on NETosis, inflammation and neointimal hyperplasia were then evaluated. Briefly, FeMOFs nanoparticle modified PLA were prepared by spin-coating. Then, PLA/FeMOFs samples were loaded with CO by pressurization. Subsequently, the physicochemical properties and CO release behavior of PLA/FeMOFs/CO were characterized. The effects of CO released from PLA/FeMOFs/CO on NETosis, macrophage behavior and endothelial cell behavior in a simulated inflammatory environment were evaluated *in vitro*. In addition, the hemocompatibility of PLA/FeMOFs/CO was also investigated. Furthermore, *in vivo* implantation in rats was further evaluated the efficacy of PLA/FeMOFs/CO.

## Materials and methods

### Materials

Ferrous chloride tetrahydrate (FeCl_2_·4H_2_O), benzene-1,3,5-tricarboxylic acid (1,3,5-BTC), Lipopolysaccharide (LPS), Phorbol 12-myristate 13-acetate (PMA) were purchased from Sigma-Aldrich Co. Ltd Sodium hydroxide (NaOH) and Dimethyl sulfoxide (DMSO) were purchased from Macklin reagent Co. Ltd Trichloromethane was purchased from Chengdu Kolon Chemical. PLA was purchased from Aladdin Co. Ltd CO gas (99.9%) was purchased from Chengdu Shimao Gas Co. Ltd SYTOX green Nucleic Acid Stain was purchased from Maokang Bio Co. Ltd Hoechst33342, Rhodamine were purchased from Beyotime Co. Ltd Mouse bone marrow neutrophil kit was purchased from Solarbio Co. Ltd FITC anti-mouse CD11b Antibody, PE anti-mouse Ly-6G Antibody, tumor necrosis factor-α (TNF-α) ELISA Kit, Interleukin-10 (IL-10) ELISA Kit were purchased from Biolegend Co. Ltd Mouse NE ELISA Kit, Mouse NETs ELISA Kit were purchased from Jianglaibio. Cell Counting Kit-8 (CCK-8) was purchased from DOJINDO. All chemicals and reagents were commercially available and used without further purification.

Male Sprague-Dawley rats (8 weeks) and New Zealand White rabbits (2 kg) were purchased from Chengdu Dashuo Laboratory Animal Co. All mice were housed in a dedicated animal room at Sichuan University. All animal experiments were conducted in accordance with the regulations of the Animal Ethics Committee of Southwest Jiaotong University (SWJTU-2103-007, NSFC) and complied with institutional ethical regulations and guidelines on animal welfare.

### Characterization

The microstructure of samples was observed by field emission scanning electron microscopy (SEM, JSM 7800F Prime). The crystal structures of samples were characterized by X-ray diffraction (XRD, Malver Panalytical, Empyrean). The functional groups of samples were recorded by Fourier transform infrared spectrometer (NICOLET5700FT-IP). The water contact angles of samples were measured using a contact angle instrument (DSA100). Flow cytometry was performed using Beckman Coulter flow cytometer. Immunofluorescence imaging was performed using a fluorescence microscope (OLYMPUS DP80).

### Sample preparation and CO loading

FeMOFs nanoparticles were synthesized according to the previous method of our groups. Detailed preparation methods are provided in the Supplementary Information. In order to obtain a uniformly mixed hybrid material, a spin coating method was used and it was carried out on stainless steel foil. Stainless steel foil was cut into squares (1 cm × 1 cm), and was ultrasonically cleaned sequentially in ethanol and water. First, a chloroform solution of 2% PLA was prepared and 2 mg/ml of FeMOFs nanoparticles were added and stirred at room temperature for 24 h to obtain a mixed solution of PLA and FeMOFs. Then, stainless steel foil was placed on a spin coater and the prepared mixing solution was spin-coated onto the surface, the obtained samples were labeled as PLA/FeMOFs, and the samples containing only PLA solution were labeled as PLA.

CO gas loading was performed through a customized CO loading device. The PLA/FeMOFs samples were placed into the CO loading device, and the system was first kept in a vacuum state and then pressurized with 1 MPa of CO gas for 12 h. Then the reactor was flushed with nitrogen and the obtained samples were labeled PLA/FeMOFs/CO.

### CO release of PLA/FeMOFs/CO

The CO release of PLA/FeMOFs/CO was detected using a Nile Red-Pd fluorescent probe (1-Ac). 400 μl DMSO, 20 μl 1-Ac (0.15 mg/ml) and 600 μl PBS were added into samples sequentially, and then incubated in an oscillator at 37°C for 24 h in darkness. Finally, they were detected using a fluorescence spectrophotometer. For the sustained duration detection, the PLA/FeMOFs/CO were incubated in PBS for 1 day, 2 days, and 3 days, respectively, and after that the retained CO release was analyzed as described above.

### Evaluation of NETosis *in vitro*

Detailed information on the extraction and characterization of neutrophils are provided in the Supplementary Information. The UV-sterilized samples were placed in 24-well plates, then freshly extracted neutrophils were seeded on the samples with a density of 2 × 10^5^ cells/ml and cultured with 20 ng/ml PMA or 1 μg/ml LPS. After being cultured for 24 h, the cells were washed with PBS and fixed with paraformaldehyde for 1 h, stained with 0.5 μM SYTOX green for 1 h, and counterstained with Hoechst33342. After washing with PBS, the cells were observed under a fluorescence microscope. To quantify the amount of NETs and NE produced by PMA-stimulated neutrophils *in vitro*, cell supernatants were collected after 24 h of culture and assayed by mouse NETs ELISA kit and mouse NE ELISA kit. For ROS assay, samples were incubated with PMA-stimulated cells for 24 h. The cells were washed with PBS, stained with 10 μM DCFH-DA for 30 min. Subsequently, samples were visualized by fluorescence microscopy. The average fluorescence intensity of each sample was measured using Image Pro Plus.

### Evaluation of macrophages *in vitro*

The UV-sterilized samples were placed in 24-well plates, then macrophages were seeded on the samples with a density of 5 × 10^4^ cells/ml and cultured with 1 μg/ml LPS. After being cultured for different time intervals, the cells were washed with PBS and fixed with 2.5% glutaraldehyde, stained by rhodamine and visualized by fluorescence microscope. Meanwhile, in order to quantify the amount of TNF-α and IL-10 factors expressed in each sample, cell supernatants were collected after 1 day culture and assayed using the Mouse IL-10 ELISA kit and Mouse TNF-α ELISA kit.

### Evaluation of human umbilical vein endothelial cells *in vitro*

The UV-sterilized samples were placed in 24-well plates, then human umbilical vein endothelial cells (HUVECs) were seeded on the samples with a density of 1 × 10^4^ cells/ml. After being cultured for 48 h. The media were replaced with culture medium containing 10% CCK-8, and the cell activity was determined by a microplate reader. Then, the cells were washed with PBS and fixed with 2.5% glutaraldehyde, stained by rhodamine, and visualized by fluorescence microscope.

HUVECs culture in a simulated inflammatory microenvironment. HUVECs were co-cultured with inflammation-stimulated neutrophil or macrophage supernatants to mimic the inflammatory environment of HUVECs *in vivo*, and to evaluate the effect of samples on the growth behavior of inflammation-stimulated HUVECs. First, the medium of different samples co-culture with 20 ng/ml PMA-stimulated neutrophils or co-culture with 1 µg/ml LPS-stimulated macrophages after 1 day were collected, respectively. The collected medium was centrifuged at 1500 r for 5 min, and the supernatants obtained were mixed with F12 medium containing 10% serum in a ratio of 3:8 as culture medium for inflammation-stimulated HUVECs experiment. Then, UV-sterilized samples were placed in 24-well plates, HUVECs were seeded with a density of 1.5 × 10^4^ cells/ml with corresponding conditioned culture media. After being cultured for different time intervals (24, 48 h). The media were replaced with culture medium containing 10% CCK-8, and the cell activity was determined by a microplate reader. Then, the cells were washed with PBS and fixed with 2.5% glutaraldehyde, stained by rhodamine and visualized by fluorescence microscope.

### Evaluation of the hemocompatibility *in vitro*

The hemolysis rate of the samples was evaluated. Fresh anticoagulated blood was obtained from New Zealand White rabbits. Fresh whole blood was diluted to 2% of concentration with saline. The samples were placed in 24-well plates and diluted whole blood was added to submerge the samples. Positive control was fresh whole blood diluted to 2% with deionized water and negative control was fresh whole blood diluted to 2% with saline. All samples were incubated in a shaker at 37°C for 1 h. The reacted blood was loaded into new centrifuge tubes centrifuged at 1000 rpm for 10 min, the supernatant was aspirated, and the absorbance value was determined by an enzyme meter at 545 nm, and the hemolysis rate was calculated.

Platelet adhesion and activation were evaluated. Fresh whole blood was centrifuged at 1500 rpm for 15 min to separate the hemocytes to obtain platelet-rich plasma (PRP), and then 100 μl of PRP was added dropwise to the surface of each sample and incubated for 30 min in a shaker at 37°C. Subsequently, the samples were washed with PBS and fixed overnight in paraformaldehyde. The fixed samples were dehydrated in a gradient of 50%, 75% and 100% alcohol and dried, followed by SEM to observe the activation status of platelets.

### *In vivo* implantation

Eight-week-old male Sprague-Dawley rats were divided into 3 groups, namely, PLA, PLA/FeMOFs and PLA/FeMOFs/CO (*n* = 3 per group). PLA and PLA/FeMOFs were prepared by dip-coating and PLA/FeMOFs/CO were prepared by loading CO in the same way as before. Rats were anesthetized and the prepared samples were implanted into the abdominal aorta. After 30 d of implantation, the vessels were removed at the sample location, rinsed with saline, fixed with 4% paraformaldehyde overnight, and sectioned by paraffin embedding. Soft tissue sections were stained with HE, MPO, CD31 immunofluorescence staining, and CD68/CD163 double immunofluorescence staining.

### Statistical analysis

One-way ANOVA was used for statistical analysis. Value of *P ≤ 0.05, **P ≤ 0.01, ***P ≤ 0.001 was statistically significant. All data are presented as mean ± SD.

## Results

### Surface characterization

**Figure 2.**
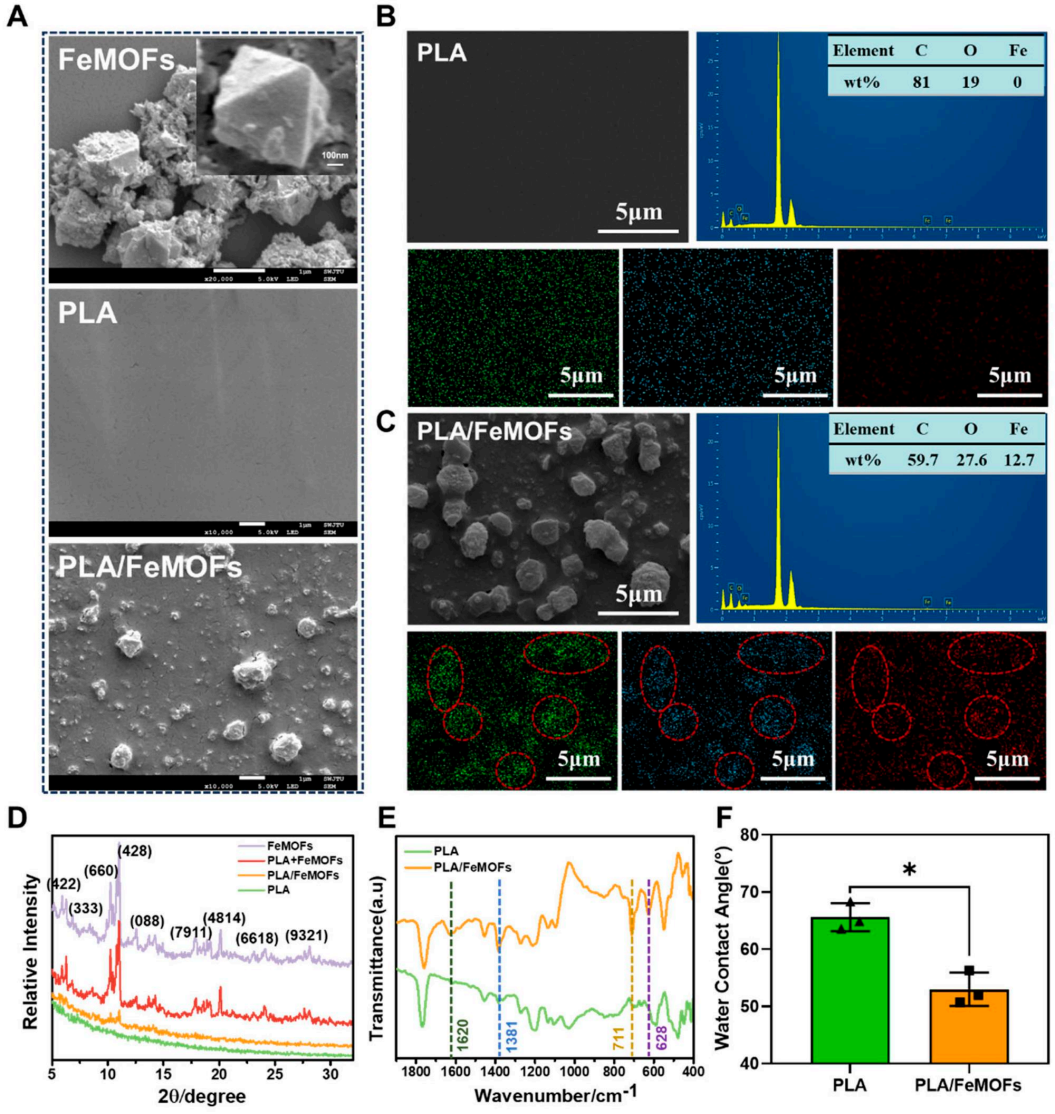
Materials characterization of PLA/FeMOFs/CO. (**A**) SEM images of FeMOFs nanoparticle, PLA and PLA/FeMOFs. EDS of (**B**) PLA and (**C**) PLA/FeMOFs. (**D**) XRD pattern of FeMOFs nanoparticle, PLA+FeMOFs Co-mingled particles, PLA and PLA/FeMOFs. (**E**) FT-IR spectra of PLA and PLA/FeMOFs. (**F**) Water contact angles of PLA and PLA/FeMOFs. Data are presented as means ± SD and analyzed using one-way ANOVA, *P < 0.05, **P < 0.01, ***P < 0.001.

FeMOFs nanoparticles were prepared by simple aqueous phase synthesis at room temperature. The prepared nanoparticles were incorporated in PLA by spin-coating. The SEM results showed that FeMOFs nanoparticles had a regular octahedral crystal structure. The prepared PLA samples were flat and smooth, and surface roughness obviously increased after FeMOFs incorporation. It can be seen that FeMOFs were dispersed in PLA with some in the shallow surface (Figure 2A). To further analyze the distribution of FeMOFs nanoparticles on the samples, energy-dispersive spectra (EDS) analysis was performed (Figure 2B and C), which showed a significant increase of Fe element of PLA/FeMOFs (12.7%), compared to that of the PLA (0%). Pseudo staining of C, O and Fe signal showed colocalization of FeMOFs nanoparticles. Uniform distribution of Fe signal indicated that FeMOFs were relatively uniformly distributed in the coating.

The XRD results showed that the prepared FeMOFs nanoparticles was a typical MIL-100(Fe) crystal structure. To explore the stability of FeMOFs in chloroform solvent, the nanoparticles and PLA were incubated in chloroform for 24 h, and then the crystal structure was examined. It showed the crystal structure did not change compared with that of the untreated FeMOFs nanoparticles (Figure 2D). Subsequently, the crystal structures of the samples were examined, there was no diffraction peaks appeared on the PLA samples, while some strong diffraction peaks of FeMOFs existed on PLA/FeMOFs samples, indicating that FeMOFs nanoparticles were successfully incorporated to PLA coating and the crystal structure did not change.

In addition, the surface chemical compositions of PLA and PLA/FeMOFs samples were characterized by Fourier infrared spectra (Figure 2E). Compared with PLA, the characteristic peaks of benzene at 1620 cm^−1^, Fe-OH at 1381 cm^−1^, Fe-O bond at 628 cm^−1^ were observed on PLA/FeMOFs.

The water contact angle was 65.6° for sample PLA and 52.9° for sample PLA/FeMOFs (Figure 2F). The positively charged iron ions and negatively charged ligands of FeMOFs increased the surface hydrophilicity.

### CO release of PLA/FeMOFs/CO

**Figure 3.**
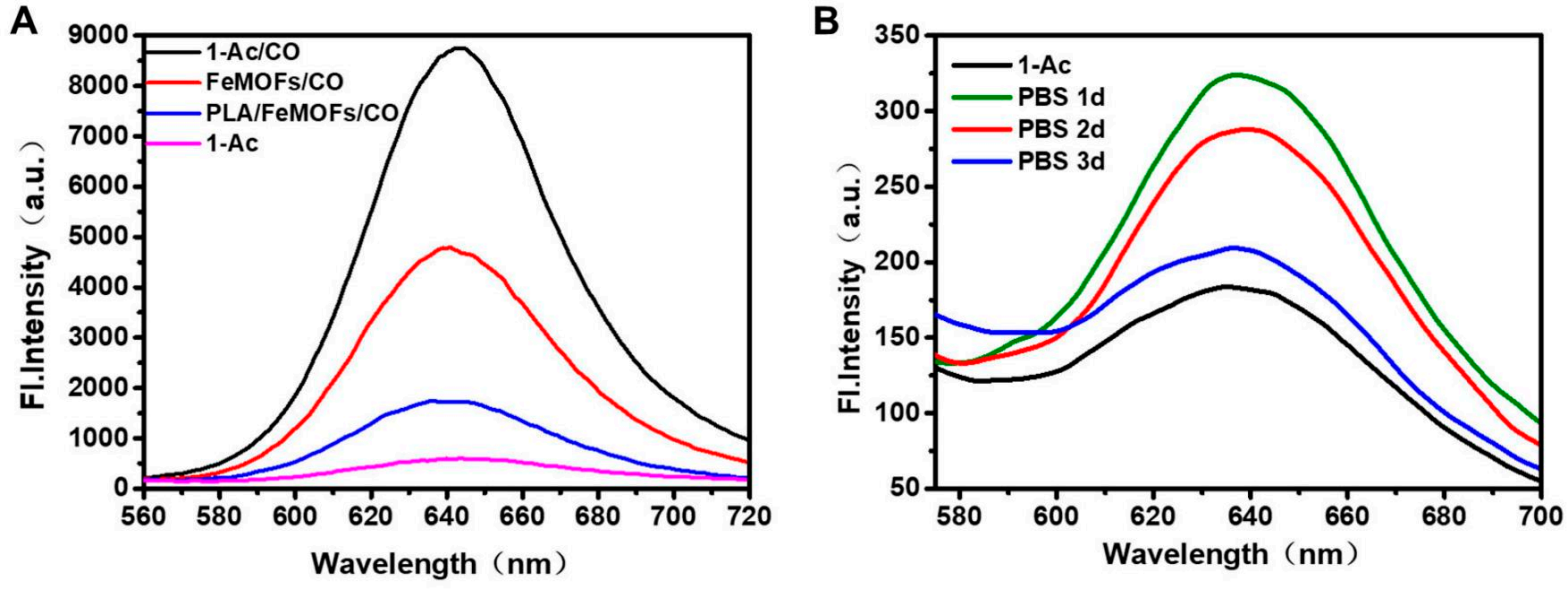
CO releasing detected by CO fluorescent probe 1-Ac. (**A**) Fluorescence spectra of monitor CO release from 1-Ac/CO, FeMOFs/CO, PLA/FeMOFs/CO. (**B**) Fluorescence spectra of monitor CO release from PLA/FeMOFs/CO after PBS incubation.

CO release of PLA/FeMOFs/CO was detected using a CO fluorescent probe 1-Ac. When CO was released from the samples, palladium (II) would bind to CO, which led to dissociation of 1-Ac and freeing of fluorescent Nile Red. CO gas was used as the positive control, and it showed that the fluorescence intensity was significantly enhanced when CO was added in 1-Ac. Compared with FeMOFs/CO, the fluorescence intensity of PLA/FeMOFs/CO significantly decreased (Figure 3A). It may be because the amount of CO loading dropped when FeMOFs incorporated in PLA, and also the release may slow down with PLA wrapping. To investigate the duration of CO release, PLA/FeMOFs/CO was incubated in PBS for 1 day, 2 days, and 3 days, respectively, and then the remained CO release was detected. The results showed that the remained CO release decreased with the incubation time. But there was still an increased fluorescence intensity compared to that of the negative control (Figure 3B), suggesting that the duration of CO release of PLA/FeMOFs/CO sample was at least 3 days.

### Inhibition NETosis of PLA/FeMOFs/CO *in vitro*

**Figure 4.**
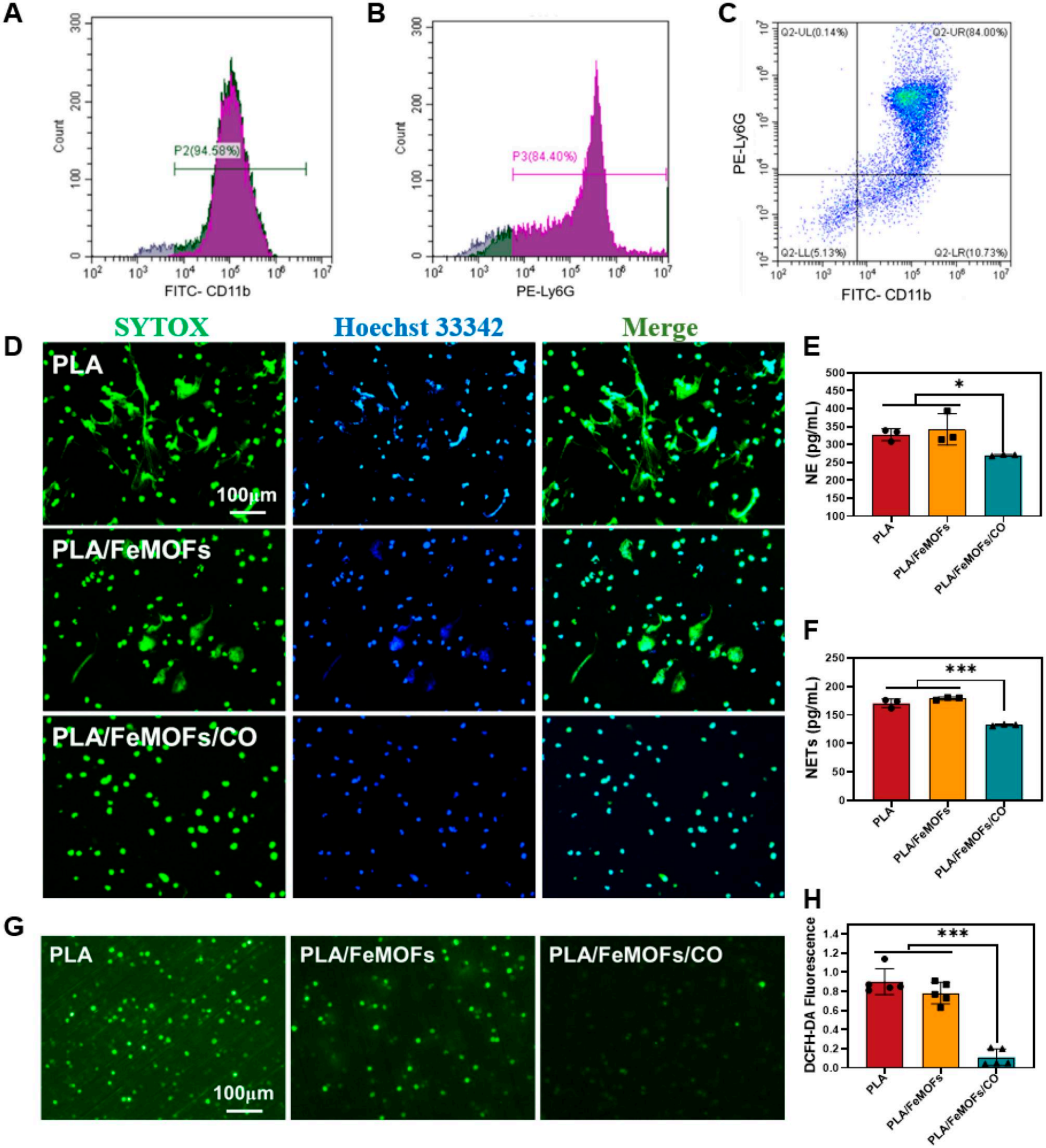
NETosis Inhibition function of PLA/FeMOFs/CO *in vitro*. (**A**) FITC-CD11b, (**B**) PE-Ly6G, (**C**) FITC-CD11b and PE-Ly6G stains to determine the purity of extracted neutrophils by flow cytometry. (**D**) Fluorescence images of NETs stained with SYTOX and Hoechst33342 by PMA induced neutrophils. (**E**) NE and (**F**) NETs concentration of PMA induced neutrophils quantified by ELISA. (**G**) Fluorescence images of PMA induced neutrophils stained with DCFH-DA. (**H**) The quantification results of fluorescence intensity. Data are presented as means ± SD and analyzed using one-way ANOVA, *P < 0.05, **P < 0.01, ***P < 0.001.

Neutrophils were isolated and extracted from mouse bone marrow, and then were identified. Phagocytic-like cells including monocytes, macrophages, neutrophils and NK cells were labeled with the FITC anti-mouse CD11b Antibody (Figure 4A), and neutrophils were specifically labeled with PE anti-mouse Ly-6G Antibody (Figure 4B), and flow cytometry analysis showed the extracted purity of neutrophils was 84% (Figure 4C).

PMA and LPS were used to induce neutrophils to NETosis and to simulate inflammation. The SYTOX green was used to stain the extracellular DNA and to show the NETs morphology. For PMA inducing group, obvious filamentous and crosslinking structures were seen on PLA and PLA/FeMOFs samples, indicating NETs release on the two samples. Whereas no filamentous NETs were seen on the PLA/FeMOFs/CO (Figure 4D). There was a similar result in the LPS induced group (Supplementary Figure S1).

The levels of NE and NETs on different samples were further quantitatively measured by ELISA kits. It showed that there were no significant difference of NE and NETs release between PLA and PLA/FeMOFs. However, PLA/FeMOFs/CO significantly reduced the release of NE and NETs (Figure 4E and F). DCFH-DA was used for evaluation of intracellular ROS generation. A bright green fluorescence was observed on PLA and PLA/FeMOFs samples, and a significant weak green fluorescence was observed on PLA/FeMOFs/CO samples (Figure 4G and H), suggesting that PLA/FeMOFs/CO significantly reduced the intracellular ROS. All the above results suggested that CO release of PLA/FeMOFs/CO significantly inhibited NETs release and NE expression by reducing intracellular ROS.

### Anti-inflammatory function of PLA/FeMOFs/CO *in vitro*

**Figure 5.**
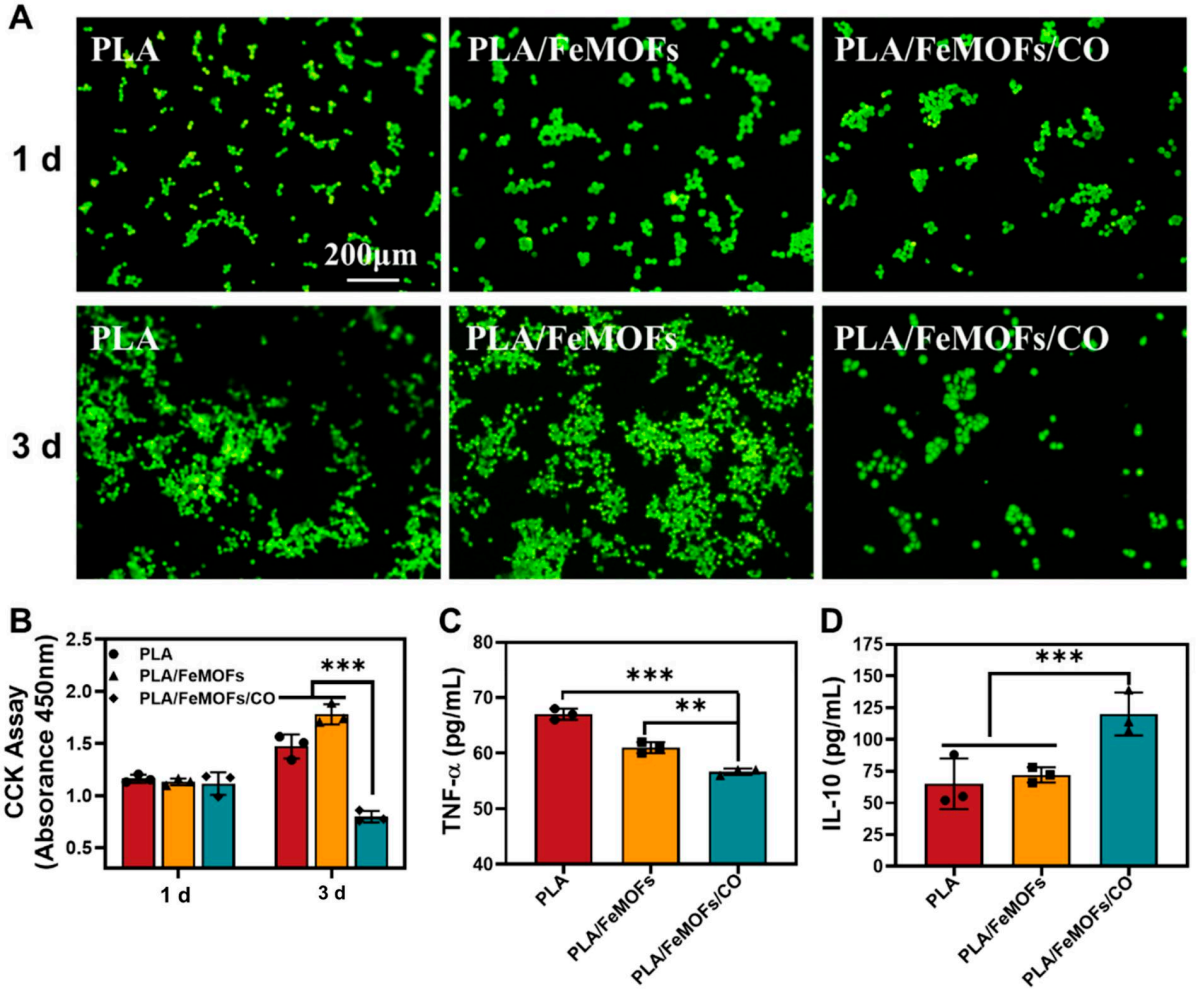
The anti-inflammatory function of PLA/FeMOFs/CO *in vitro*. (**A**) Rhodamine staining of macrophages on samples after 1 day and 3 days culture. (**B**) CCK-8 assay of macrophages on samples after 1 day and 3 days culture. (**C**) TNF-α and (**D**) IL-10 concentration of macrophages quantified by ELISA. Data are presented as means ± SD and analyzed using one-way ANOVA, *P < 0.05, **P < 0.01, ***P < 0.001.

The LPS-induced macrophage inflammation model was chosen to observe the anti-inflammatory function of PLA/FeMOFs/CO. There was no significant difference of samples on macrophage adhesion at day 1. After incubation for 3 days, cell proliferation on PLA and PLA/FeMOFs were increased, while that on PLA/FeMOFs/CO were significantly reduced. Meanwhile, the morphology of macrophages adhered on PLA/FeMOFs/CO showed a decreased activated state with round shape, but that on PLA and PLA/FeMOFs showed high activated shuttle shape (Figure 5A). Both the Rhodamine staining and CCK-8 assay (Figure 5A and B) results suggested that PLA/FeMOFs/CO reduced macrophage proliferation and maintained its low activated state.

Subsequently, the samples were cocultured with LPS-induced macrophages for 24 h, and then the supernatants were collected for inflammatory factor analysis. Typical proinflammatory factor TNF-α and anti-inflammatory factor interleukin-10 (IL-10) were evaluated. There was no significant difference in TNF-α and IL-10 expression between PLA and PLA/FeMOFs. It showed PLA/FeMOFs/CO significantly inhibited TNF-α expression and promoted IL-10 expression compared with the other two groups (Figure 5C and D).

### HUVECs growth behavior in simulated inflammatory model *in vitro*

Endothelialization formation plays an important role for vascular stent implant materials. Therefore, the endothelial cell adhesion behavior of different samples was evaluated. The results of fluorescence photography and CCK-8 assay (Supplementary Figure S2) showed that there was a decrease cell viability in PLA/FeMOFs compared to PLA after 2 days of culture, but cell viability of PLA/FeMOFs/CO was not affected, and was significantly increased than compared to PLA/FeMOFs group. It showed that PLA/FeMOFs/CO maintained cell viability of HUVECs due to the release of CO.

**Figure 6.**
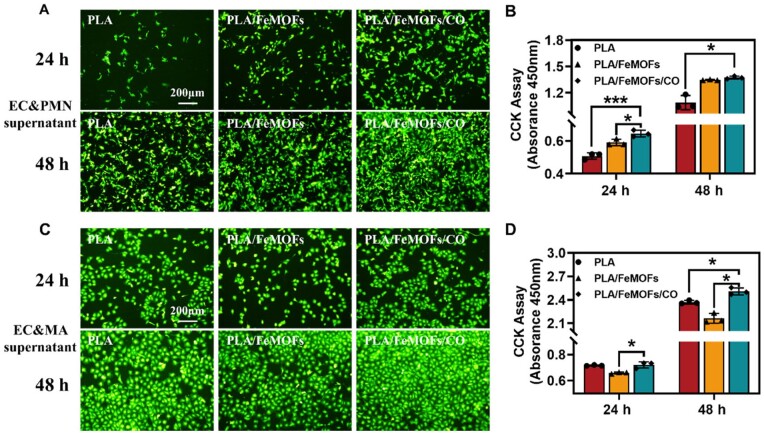
HUVECs Growth behavior in simulated inflammatory microenvironment. (**A**) Rhodamine staining of HUVECs after 24 and 48 h of culture with neutrophil conditioned medium. (**B**) CCK-8 assay after 24 and 48 h of culture with neutrophil conditioned medium. (**C**) Rhodamine staining of HUVECs after 24 and 48 h of culture with macrophage conditioned medium. (**D**) CCK-8 assay after 24 and 48 h of culture with macrophage conditioned medium. Data are presented as means ± SD and analyzed using one-way ANOVA, *P < 0.05, **P < 0.01, ***P < 0.001.

Due to the inflammatory response of PLA stent materials in service, neutrophils are activated to release NETs and macrophages are polarized to a typical M1 pro-inflammatory phenotype, thereby inhibiting endothelial cell growth. Therefore, we further explored the effects of modulating the inflammatory microenvironment on endothelial cell growth behavior. PLA, PLA/FeMOFs, PLA/FeMOFs/CO were, respectively, co-cultured with HUVECs for 24 and 48 h in each neutrophil conditioned medium. For each neutrophil-conditioned medium, the three samples were, respectively, cultured with PMA-treated neutrophils and then different inflammatory mediators were in each conditioned medium due to different material interfering. The results of fluorescence photography (Figure 6A) showed that morphology of endothelial cells on samples shifted from paving stone to long spindle morphology, it suggested that inflammatory stimulation was produced in all samples. Specifically, PLA/FeMOFs/CO had reduced long shuttle transition compared to PLA and PLA/FeMOFs, which indicated attenuated inflammatory stimulation. And CCK-8 assay (Figure 6B) showed PLA/FeMOFs/CO significantly enhanced endothelial cell activity which indicated inflammatory mediators reduce in the microenvironment is beneficial for endotheliazation.

Similarly, PLA, PLA/FeMOFs, PLA/FeMOFs/CO were co-cultured with HUVECs for 24 and 48 h in each macrophage-conditioned medium. The results of fluorescence photography and CCK-8 assay (Figure 6C and D) showed that there was no significant change in cell morphology was observed in all samples, while the PLA/FeMOFs/CO samples also significantly enhanced endothelial cells activity and growth compared to the PLA and PLA/FeMOFs samples.

As described in Sections Inhibition NETosis of PLA/FeMOFs/CO *in vitro* and Anti-inflammatory function of PLA/FeMOFs/CO *in vitro*, PLA/FeMOFs/CO reduced NETs formation by PMA-treated neutrophils and reduced the proinflammatory factor TNF-α by LPS-treated macrophages. Thus, PLA/FeMOFs/CO improved the neutrophil and macrophage-induced inflammatory microenvironment, which was in turn enhanced endothelial cell adhesion and growth.

### Evaluation of the hemocompatibility *in vitro*

**Figure 7.**
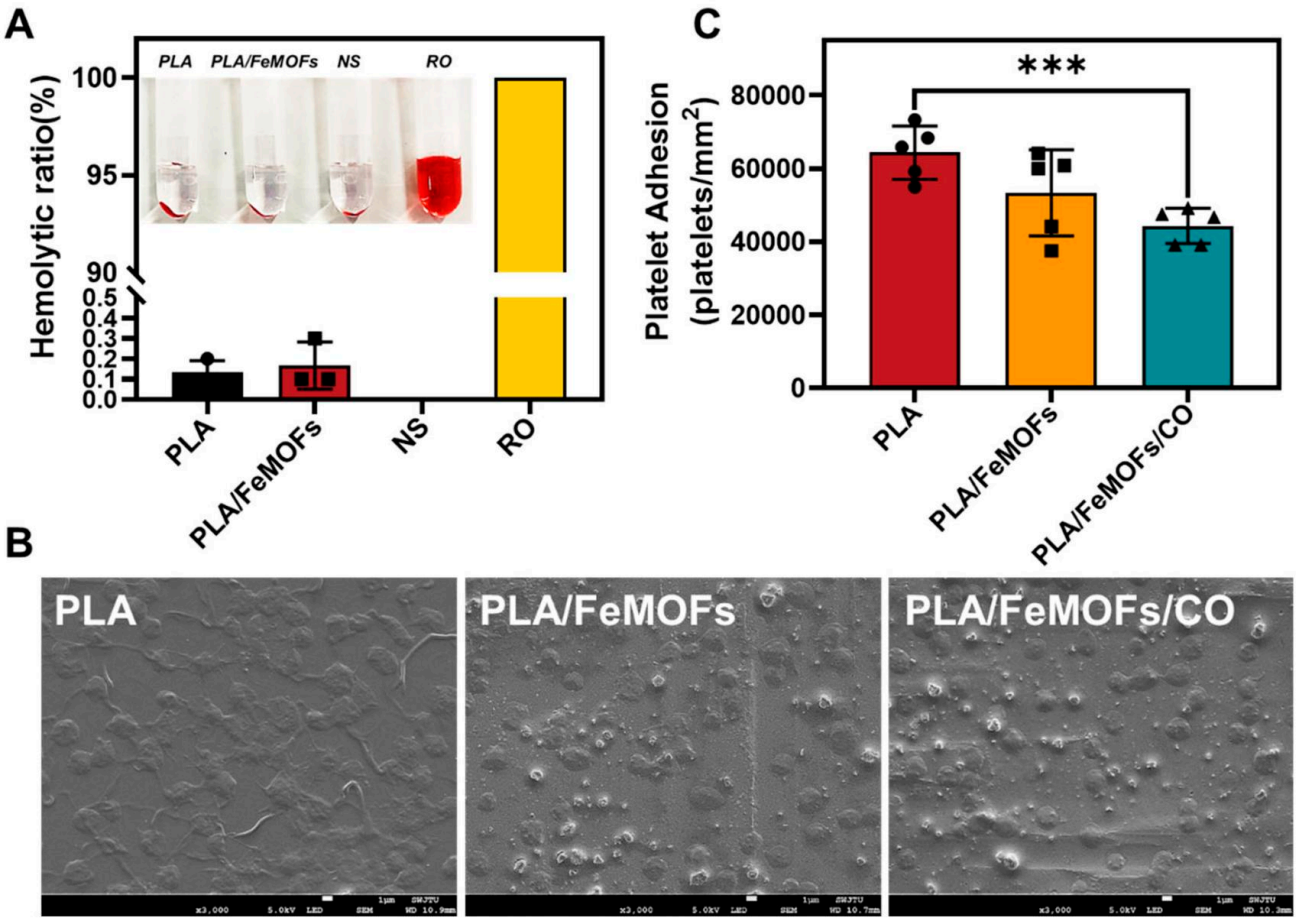
*In vitro* hemocompatibility evaluation. (**A**) Hemolysis rate of PLA and PLA/FeMOFs. (**B**) SEM observation of the platelet adhesion and activation. (**C**) Platelet adhesion level after 30 min incubation with PRP. Data are presented as means ± SD and analyzed using one-way ANOVA, *P < 0.05, **P < 0.01, ***P < 0.001.

Hemolysis is an important indicator for blood contacting biomaterials. The results of hemolysis experiments showed that the hemolysis rates of PLA and PLA/FeMOFs were less than 5% (Figure 7A), which conform the criteria for blood contacting biomaterials.

Platelet adhesion and activation will trigger the coagulation process, and it is very important to reduce platelet adhesion and inhibit platelet activation on the surface of blood contacting biomaterials. SEM results showed significant activation of platelets on PLA, and that on PLA/FeMOF was reduced. It was further reduced on PLA/FeMOF/CO (Figure 7B and C), suggesting that CO released from PLA/FeMOF/CO improved the hemocompatibility.

### *In vivo* implantation

**Figure 8.**
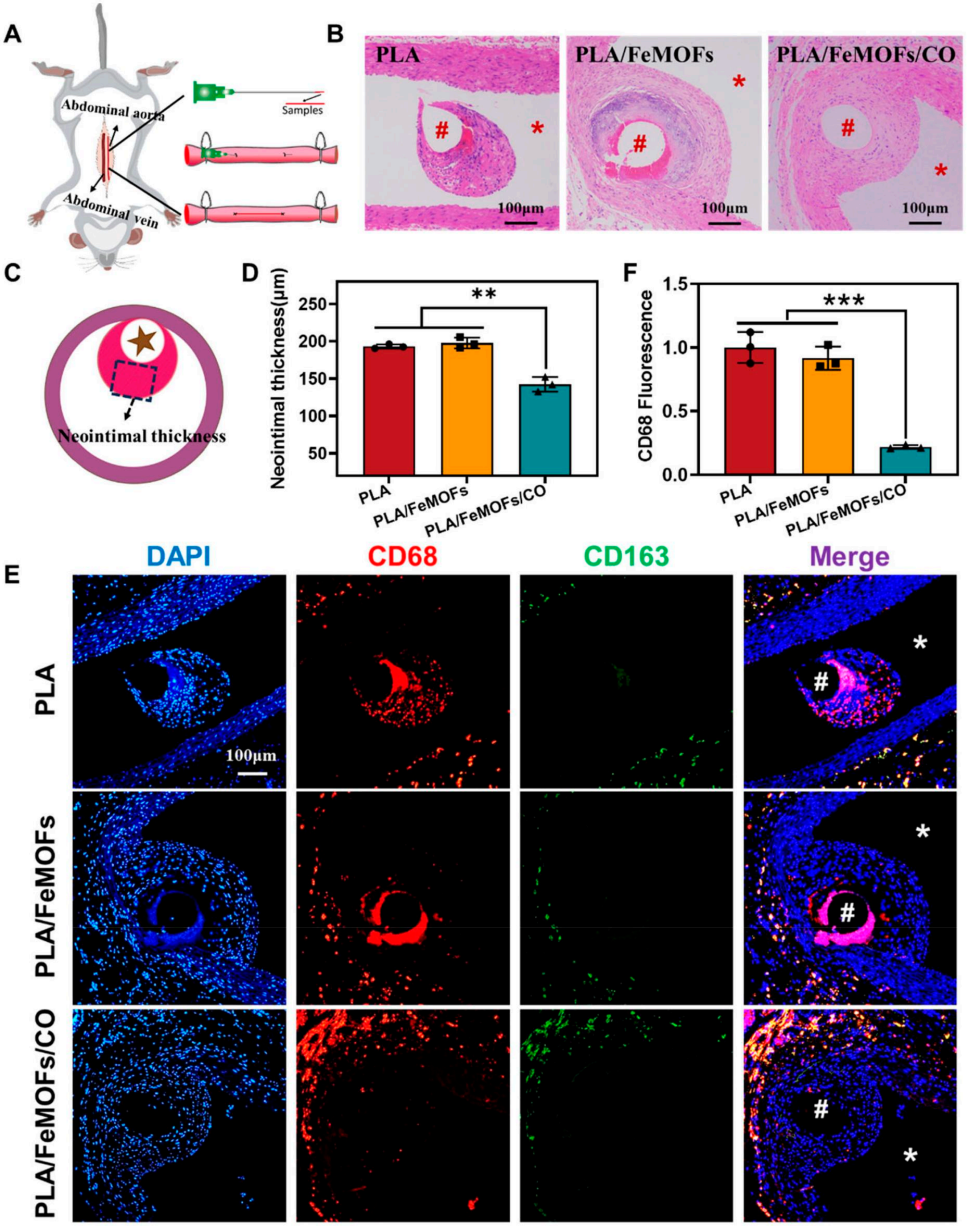
*In vivo* Sprague-Dawley rats implantation experiments. (**A**) *In vivo* implantation rat model. (**B**) Hematoxylin and eosin-stained after implantation for 30 days. (**C**) Schematic diagram of neointimal thickness measurement. (**D**) The quantification results of the neointimal tissue thickness. (**E**) Images of CD68 immunofluorescence, CD163 immunofluorescence and nuclei were stained with DAPI. (**F**) Statistics of CD68 fluorescence intensity in neointimal tissue. Data are presented as means ± SD and analyzed using one-way ANOVA, *P < 0.05, **P < 0.01, ***P < 0.001.

The efficacy of PLA/FeMOF/CO was evaluated by *in vivo* implanting (Figure 8A). The implant process and materials injured vascular wall tissues, and neointimal which encapsulated the implant materials after 30 days was evaluated. HE staining results showed that all samples were covered by neointimal tissue after 30 days of implantation (Figure 8B). For the PLA and PLA/FeMOFs samples, the neointimal tissue with white gaps and loose tissue. There was a large amount of dark blue tissue with fibrin proximal the samples, which could be mainly an inflammatory response caused by monocytes infiltration and thrombus formation. For the PLA/FeMOFs/CO samples, it showed the cell nuclei in the neointimal tissue were evenly distributed and densely organized, and the monocyte infiltration was significantly reduced. Subsequently, the thickness of neointimal tissue which was perpendicular to the implantation position of the samples was measured (Figure 8C). It showed that there was no significant difference in neointimal tissue thickness between sample PLA (193.1 ± 2.9 µm) and sample PLA/FeMOFs (197.8 ± 7.1 µm). While the neointimal tissue thickness was significantly decreased for sample PLA/FeMOFs/CO (142.4 ± 9.8 µm) (Figure 8D), indicating intimal hyperplasia was reduced.

CD68 is the marker of the proinflammatory macrophage, and CD163 is the marker of anti-inflammatory macrophage. In addition, immunofluorescence staining of CD68 and CD163 was conducted for exploring inflammatory response of the infiltrated macrophage. Positive and high expression of CD68 was seen on the PLA and PLA/FeMOFs samples, while that of PLA/FeMOFs/CO significantly decreased (Figure 8E and F). Although CD163 expression on all samples cannot be seen, much lower expression of CD68 indicated PLA/FeMOFs/CO reduced the inflammatory response.

**Figure 9.**
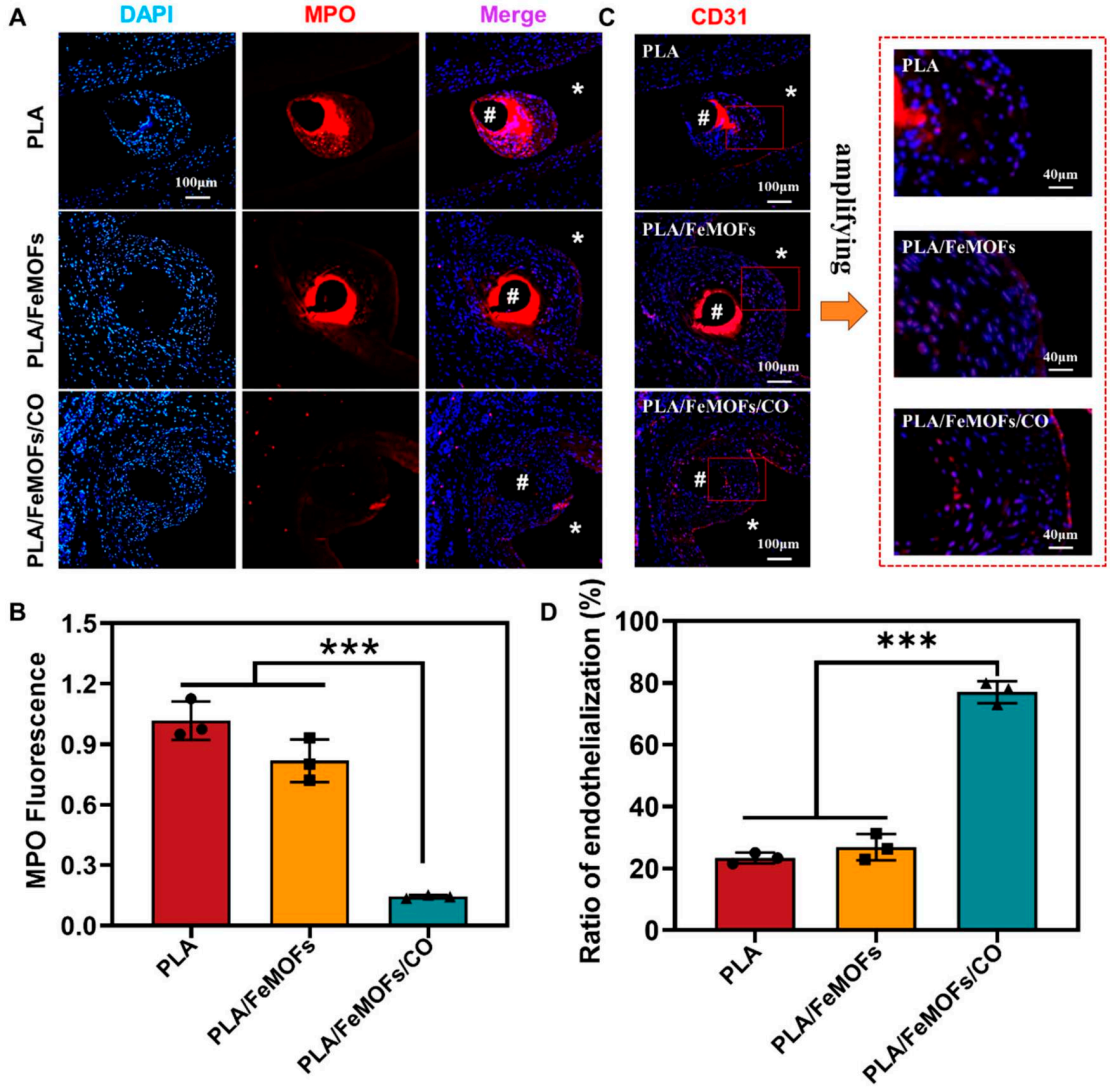
Evaluation of neutrophil infiltration and endothelialization *in vivo*. (**A**) Images of MPO immunofluorescence, and nuclei were stained with DAPI. (**B**) Statistics of MPO fluorescence intensity in neointimal tissue. (**C**) Images of CD31 immunofluorescence. The nuclei were stained with DAPI. (**D**) Quantification of endothelialization. Data are presented as means ± SD and analyzed using one-way ANOVA, *P < 0.05, **P < 0.01, ***P < 0.001.

Subsequently, neutrophil infiltration in the neointimal tissue near the samples was observed by MPO immunofluorescence staining. Positive high expression of MPO was seen on the PLA and PLA/FeMOFs samples, and essentially no MPO expression was seen on the PLA/FeMOFs/CO samples (Figure 9A and B). It indicated PLA/FeMOFs/CO samples reduced the neutrophil infiltration.

Immunostaining for the endothelial cell marker CD31 was performed to visualize re-endothelialization. Figure 9C shows incomplete endothelial cell coverage on PLA and PLA/FeMOFs samples, and a nearly complete endothelial cell coverage with CD31 expression enhanced on PLA/FeMOFs/CO samples. The endothelialization results (Figure 9D) shown PLA/FeMOFs/CO significantly increased endothelial cell coverage (77%) compared to PLA (23%) and PLA/FeMOFs (26%).

## Discussion

CO has been identified as an essential gasotransmitter with anti-inflammatory and antioxidant properties. So far, CO inhale or CORMs were used for cardiovascular disease therapy and showed some beneficial effects [28]. However, CO release duration of CORMs is very fast and can only sustain 1–2 h [31, 32]. And this limits CORMs to be directly used for cardiovascular stent modification. In this study, with FeMOFs incorporation in PLA and post loading of CO with pressurization, it showed CO release duration was at least 3 days, which can be used to reduce stenting-triggered acute phase response inflammation and regulate inflammatory microenvironment. Sustained CO release duration in this strategy was likely attributed to the affinity between FeMOFs and CO and hydrophobic PLA wrapping.

The involvement of leukocytes, such as neutrophils, plays an important role in the host immune response to implanted biomaterials. Neutrophils adhering to plaques or injured endothelium form NETs, which form a solid-state reactor on the intimal surface, exacerbating inflammation and thrombus formation [15]. NETs were first identified by Takei *et al*. in 1996, and was determined that they are a new process of PMA-induced neutrophil cells death [33]. Brinkman named this process NETosis in 2004 [34]. PMA triggers a burst of ROS mainly through activation of protein kinase C (PKC) and NADPH oxidase, which leads to the formation of NETs [35]. NE is released from neutrophils in a ROS-dependent manner during NETs formation and plays an important role in NETosis [36]. CORM-2 have been confirmed to inhibit NADPH oxidase activity and ROS production induced by particulate matter or pseudomonas aeruginosa [37, 38]. In our study, it was manifested that CO release of PLA/FeMOFs/CO inhibited PMA-induced ROS production, decrease NE expression and reduce the formation of NETs. Meanwhile, we also found that PLA/FeMOFs/CO inhibited LPS-induced NETs formation (Supplementary Figure S1). LPS, as an endotoxin, activates the p38 MARK signaling pathway leading to massive inflammatory responses and induces NETs formation [39]. Thus, it indicated CO released of PLA/FeMOFs/CO inhibited inflammatory responses and NETs by modulating the p38 MARK signaling pathway.

Macrophages, an important class of immune cells, are responsive to the host’s inflammatory response to implanted biomaterials. Researchers have demonstrated that administration of exogenous CO can effectively inhibit LPS-activated macrophages inflammation response by modulating the p38 MARK signaling pathway [22]. Simultaneously, CO release of PLA/FeMOFs/CO inhibited LPS-induced macrophage inflammation by reducing TNF-α expression, and promoting IL-10 expression.

Thrombosis is an important factor contributing to early stenting failure. Therefore, good hemocompatibility is crucial for implantable biomaterials. CORMs have been reported to inhibit platelet aggregation and activation *in vitro* [27]. It demonstrated that CO released of PLA/FeMOFs/CO reduced platelet adhesion and activation and improved the hemocompatibility.

*In vivo* results further demonstrated that PLA/FeMOFs/CO reduced the inflammatory response of macrophage and neutrophil infiltration, promoted re-endothelialization, and significantly reduced the neointimal tissue thickness compared with PLA and PLA/FeMOFs. In conclusion, PLA/FeMOFs/CO improved the efficacy of PLA stent materials by inhibiting NETosis and macrophage inflammatory response through CO release.

## Conclusion

In this study, PLA/FeMOFs/CO was prepared and exhibited desirable CO loading and sustained release. CO release of PLA/FeMOFs/CO reduced PMA-induced ROS production, decrease NE expression, thereby inhibiting NETs formation. It also decreased the LPS-induced macrophage inflammation, promotes endothelial cell growth in inflammatory environment and inhibited platelet adhesion and activation. Both *in vitro* and *in vivo* experiments confirmed that PLA/FeMOFs/CO reduced the inflammatory response caused by neutrophils and macrophages and thus decreased the neointimal tissue thickness.

## Supplementary Material

rbae140_Supplementary_Data
