# Supplementary material for: FeMOFs/CO loading reduces NETosis and macrophage inflammatory response in PLA based cardiovascular stent materials
Source: Regen Biomater. 2024 Dec 3;12:rbae140. doi: 10.1093/rb/rbae140 (PMC11703552; doi:10.1093/rb/rbae140)
Supplement: rbae140_Supplementary_Data [file rbae140_supplementary_data.docx]

**Supplementary Information:**

**FeMOFs/CO loading reduces** **NETosis and macrophage inflammatory response in PLA based** **cardiovascular stent materials**

Yinhong Xie^b,c^, Mengchen Chi^b,c^, Xinlei Yang^b,c^, Ruichen Dong^a,b^, Ao Yang^a,b^, Antao Yin^a,b^, Yajun Weng^a,b *^

***AUTHOR ADDRESS***

^a^ Institute of Biomedical Engineering, College of Medicine, Southwest Jiaotong University, Chengdu 610031, Sichuan, China

^b^ Key Laboratory of Advanced Technologies of Materials, Ministry of Education, Southwest Jiaotong University, Chengdu 610031, China

^c^ School of Materials Science and Engineering, Southwest Jiaotong University, Chengdu 610031, China

*** Corresponding author**

Email address: [wengyj7032@swjtu.edu.cn](mailto:wengyj7032@swjtu.edu.cn)

**Contents**

1. Methods to prepare FeMOFs Nanoparticles
2. Methods of the extraction and identification of neutrophils
3. Supporting figures

**Methods to prepare FeMOFs Nanoparticles**

First, 1.2 g of NaOH was dissolved in 30mL of deionized water and stirred for 10min until the powder was completely dissolved. Then, 1.68 g of trimesic acid (1,3,5-BTC) was added and dissolved ultrasonically for 10 min to prevent agglomeration to obtain a mixed solution. Subsequently, 2.485 g of FeCl_2_·4H_2_O was dissolved in 120 mL of deionized water. A mixed solution was added dropwise into the FeCl_2_·4H_2_O solution with a syringe, and the reaction was stirred for 24 h at room temperature. The suspension was centrifuged at 3500 rpm for 5 min to obtain the product and was washed with deionized water and ethanol for 3 times, and then dried at 80 ℃ in an oven for 12 h.

**Methods of the extraction and identification of neutrophils**

Extraction of neutrophils. Male C57BL/6 mice (20-25g) were decapitated and sterilized with alcohol. The femur and tibia of both hind legs of the mice were removed, and bone marrow cavity cells were rinsed with PBS. Then the rinse solution was filtered through a 100 μM filter, and the filtered cells suspension was centrifuged at 1500 rpm for 5 min to obtain the cells. Next, neutrophil isolation was performed using the Mouse bone marrow neutrophil kit. Briefly, 4 mL of Reagent A was added to a 15 mL centrifuge tube, and then 2 mL of Reagent C was slowly superimposed on top of the liquid level of Reagent A to form a gradient interface, and then a droplet of cell suspension resuspended in PBS was added on top of the liquid level of the isolate. After that, the centrifuge tube forming the gradient liquid level was centrifuged at 2000 rpm for 30 min, to obtain the stratified cells. Subsequently, neutrophils were pipetted into a new centrifuge tube and added PBS to wash the cells 3 times to obtain neutrophils, which were cultured in 1640 medium containing 10% FBS.

Identification of neutrophils. The extracted neutrophils were resuspended and counted, and the cells were removed and divided equally into 4 portions, labeled as Ctrl group, FITC-CD11b group, PE-Ly6G group, and FITC-CD11b, PE-Ly6G double-staining group. The Ctrl group was not subject to any treatment, and was resuspended directly into PBS solution; the FITC-CD11b group was added with FITC anti-mouse CD11b Antibody and stained at 4°C for 30 min; PE-Ly6G group was added PE anti-mouse Ly-6G Antibody and stained at 4°C for 30 min; FITC-CD11b, PE-Ly6G double-stained group was added FITC anti-mouse CD11b Antibody, PE-Ly6G Antibody and stained at 4°C for 30 min. The staining was washed three times with PBS and detected by flow cytometry.


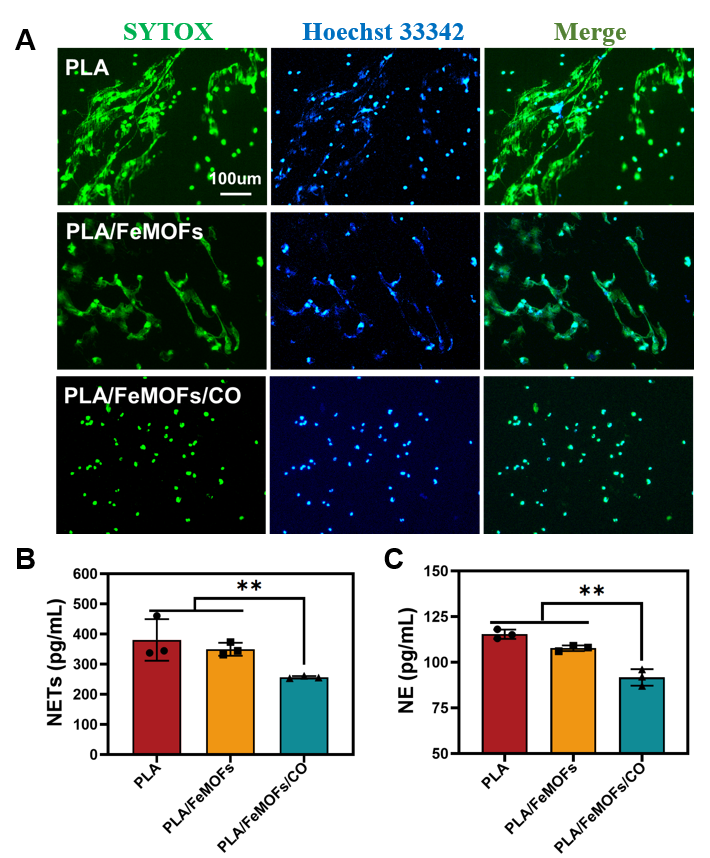


Figure S1. (A) Fluorescence images of NETs stained with SYTOX and Hoechst33342 by LPS induced neutrophils. (B) NETs and (C) NE concentration of LPS induced neutrophils quantified by ELISA. Data are presented as means ± SD (n=3) and analyzed using one-way ANOVA, **p < 0.05, **p < 0.01, ***p < 0.001*.


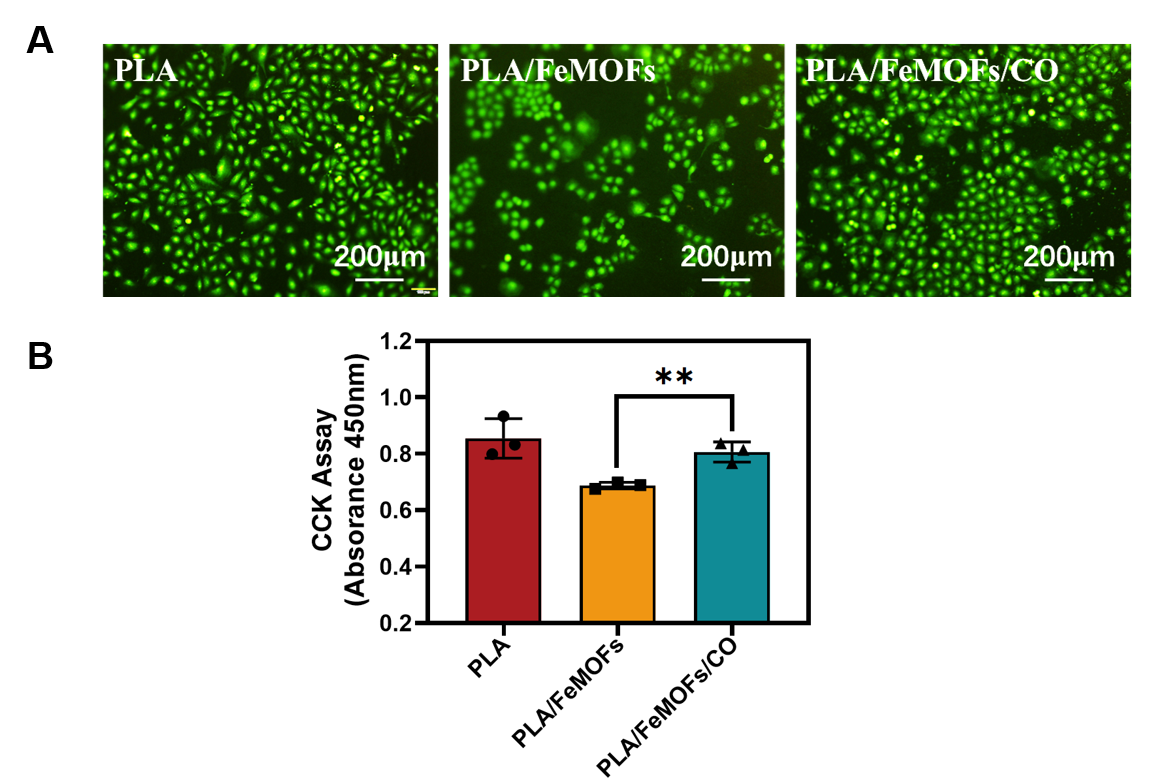


Figure S2. (A) Rhodamine staining of HUVECs after 48 h of culture. (B) CCK-8 assay after 48 h of culture. Data are presented as means ± SD (n=3) and analyzed using one-way ANOVA, **p < 0.05, **p < 0.01, ***p < 0.001*.


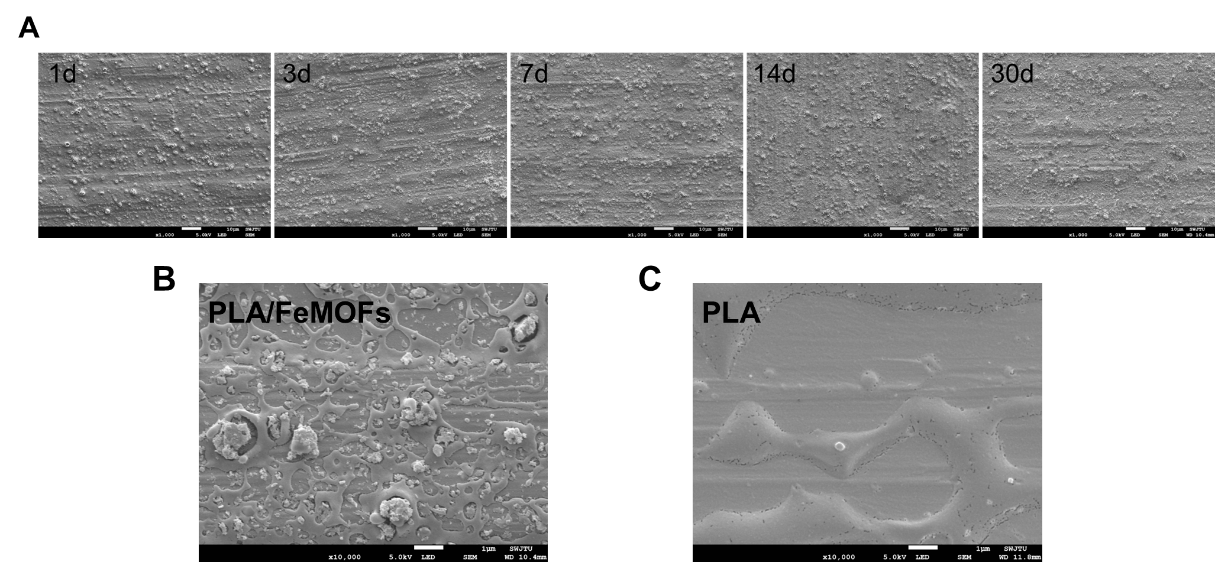


Figure S3. (A) SEM images of PLA/FeMOFs after 1 d, 3 d, 7 d, 14 d, and 30 d immersion in PBS (×1000). (B) SEM images of PLA/FeMOFs immersed in PBS for 30 d (×10000). (C) SEM image of PLA immersed in PBS for 30 d (×10000).
